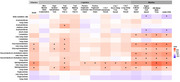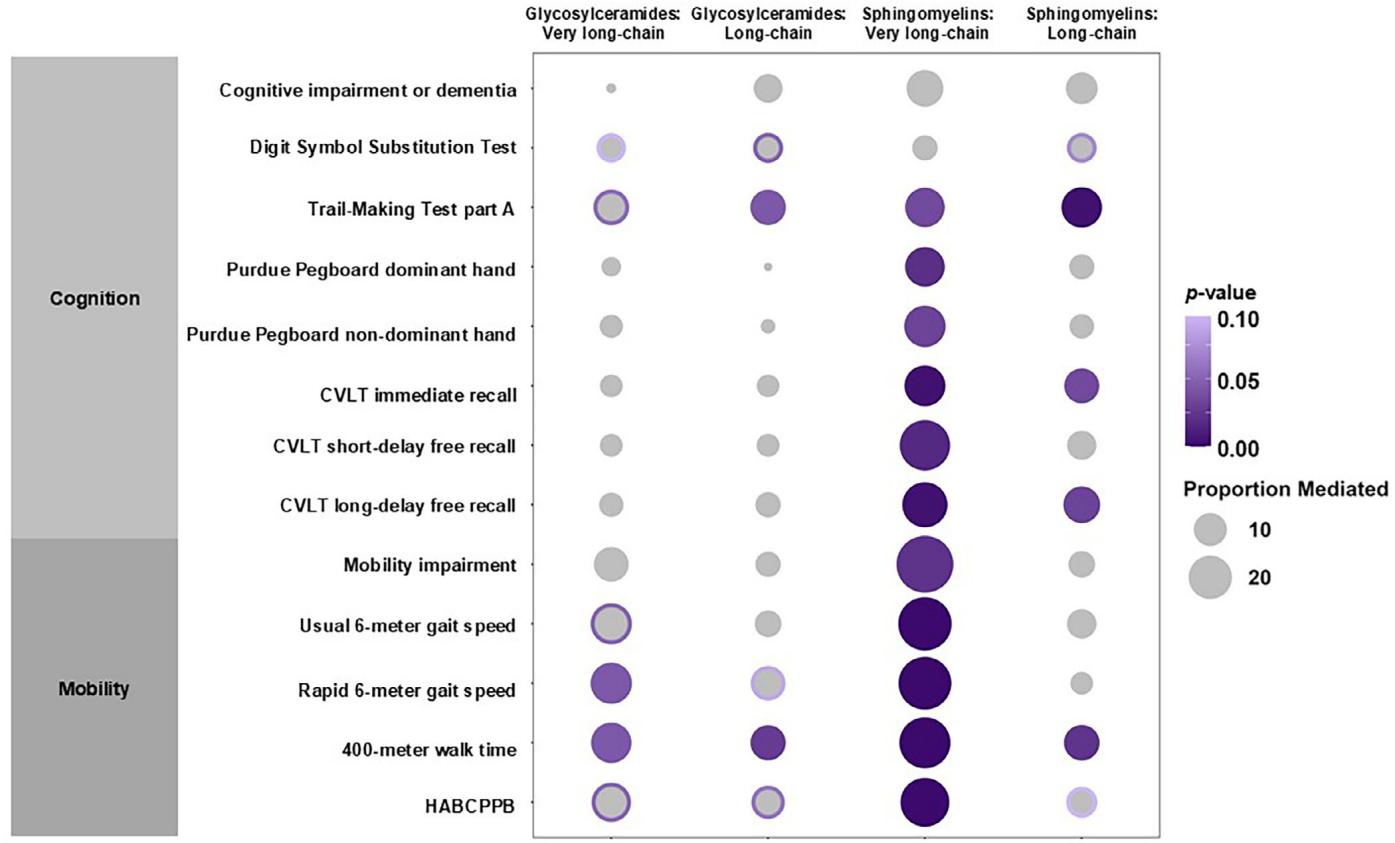# Lipid blood biomarkers mediating olfaction with cognitive and mobility impairment

**DOI:** 10.1002/alz70856_100618

**Published:** 2025-12-25

**Authors:** Qu Tian, Erin E Greig, Susan M. Resnick, Luigi Ferrucci

**Affiliations:** ^1^ National Institute on Aging, Baltimore, MD, USA; ^2^ Laboratory of Behavioral Neuroscience, National Institute on Aging Intramural Research Program, National Institutes of Health, Baltimore, MD, USA; ^3^ Translational Gerontology Branch, National Institute on Aging, NIH, Baltimore, MD, USA

## Abstract

**Background:**

Olfactory deficit is consistently associated with age‐related cognitive and mobility decline, and one of the earliest signs of neurodegenerative diseases, such as Alzheimer's disease and Parkinson's disease. However, the underlying mechanisms are not fully understood. Lipids in the nasal mucosa stabilize the olfactory nerve membrane which may contribute to olfactory transduction to the brain. The lipid composition of the nasal mucosa is likely affected by lipids in circulation. Therefore, we investigated whether plasma lipids mediated the relationships of olfaction with cognitive and mobility impairment and function, which may shed light on underlying mechanisms.

**Method:**

In 656 Baltimore Longitudinal Study of Aging participants (mean age:70.5years, 55%women, 30%Black, 3%cognitive impairment)**,** we tested the mediation effects of lipid metabolites on associations of olfaction with cognitive and mobility impairment and performance. Plasma lipids and small molecules were assayed via FIA‐ and LC‐mass spectrometry, respectively, and categorized into six lipid classes (i.e., acylcarnitines, ceramides, glycosylceramides, glycerolipids and cholesteryl esters, sphingomyelins, and triglycerides). Odor identification was scored via 16‐item Sniffin’ Sticks. Cognitive impairment was determined based on clinical characteristics, and cognitive functions were measured via a neuropsychological battery. Mobility impairment and performance were measured by gait speed. All models were adjusted for demographics and additionally adjusted for visceral fat via CT in a subsample (*n* = 570).

**Result:**

Only very long‐chain and long‐chain glycosylceramides and sphingomyelins were positively associated with both olfaction and cognitive and mobility outcomes (*p* <0.05)(Figure 1). Sphingomyelins significantly mediated associations of olfaction with cognitive outcomes of attention (Trail Making Test part A), manual dexterity (Purdue Pegboard), and verbal memory (California Verbal Learning Test), and mobility impairment and performance of usual and rapid gait speed, 400‐meter walk time, and HABCPPB up to 29.4% (all *p* <0.05). Glycosylceramides also mediated the association with attention, rapid gait speed, and 400‐meter time up to 11% (all *p* <0.05)(Figure 2). The mediating effects of sphingomyelins persisted after further adjusting for visceral fat.

**Conclusion:**

Certain lipid species of sphingomyelins and glycosylceramides mediate the relationship of olfaction with cognition and mobility. Future longitudinal studies are needed to confirm these cross‐sectional findings and further investigate the mediation role of lipid metabolism in the nasal mucosa.